# Predictors of depression, anxiety, and overall psychological distress in people living with HIV/AIDS: analyses from the *Stigma Index Brazil 2.0*

**DOI:** 10.1590/0102-311XEN021925

**Published:** 2025-11-07

**Authors:** Guilherme Welter Wendt, Angelo Brandelli Costa

**Affiliations:** 1 Programa de Pós-graduação em Ciências Aplicadas à Saúde, Universidade Estadual do Oeste do Paraná, Francisco Beltrão, Brasil.; 2 Pontifícia Universidade Católica do Rio Grande do Sul, Porto Alegre, Brasil.; 3 John Cabot University, Rome, Italy.

**Keywords:** Depression, Anxiety, Risk Factors, Depressão, Ansiedade, Fatores de Risco, Depresión, Ansiedad, Factores de Riesgo

## Abstract

Internalized stigma has been strongly linked to mental health issues in people living with HIV/AIDS (PLHA), particularly depression. Nonetheless, the overlap between depressive symptoms and other psychopathologies is well-known among specialists. This study aimed to examine the factors predicting depressive and anxiety symptoms in PLHA. This is a community-based study (*Stigma Index Brazil 2.0*) that involved 1,784 PLHA recruited from seven Brazilian state capitals. Outcomes of interest were derived from the *Patient Health Questionnaire* (PHQ), and covariates included factors previously linked to poor mental health outcomes, such as social vulnerability, treatment adherence, internalized stigma, among others identified in the *Stigma Index* study. Internalized stigma was significantly associated with worse mental health outcomes across all models (i.e., depression, anxiety, and total PHQ-4 score). Moreover, other variables were differentially associated with each outcome, possibly indicating distinct pathways with which healthcare professionals might address the burden of mental suffering in PLHA. Clear implications for public policies are equally outlined.

## Abstract (pt)

O estigma internalizado é altamente associado a problemas de saúde mental em pessoas vivendo com HIV/aids (PVHA), especialmente depressão. No entanto, a sobreposição entre sintomas depressivos e outras psicopatologias é bem conhecida pelos especialistas. Portanto, este estudo teve como objetivo examinar os fatores que predizem sintomas depressivos e ansiosos em PVHA. Trata-se de um estudo de base comunitária (*Índice de Estigma Brasil 2.0*) que envolveu 1.784 PVHA recrutadas em sete capitais brasileiras. Os desfechos de interesse foram derivados do *Questionário de Saúde do Paciente* (QSP). As covariáveis foram aquelas que foram previamente associadas a desfechos desfavoráveis, como vulnerabilidade social, adesão ao tratamento, estigma internalizado, entre outros eleitos no estudo *Índice de Estigma*. O estigma internalizado foi significativamente associado a piores desfechos em saúde mental em todos os modelos (i.e., depressão, ansiedade e pontuação total do QSP-4). Além disso, outras variáveis foram diferencialmente associadas a cada desfecho, possivelmente indicando caminhos pelos quais os profissionais de saúde podem abordar o fardo do sofrimento mental em PVHA. Implicações claras para políticas públicas são igualmente delineadas.

## Abstract (es)

El estigma internalizado se ha relacionado estrechamente con problemas de salud mental en personas con VIH/SIDA (PVVS), especialmente con la depresión. Sin embargo, como bien saben los especialistas, existe una conexión entre los síntomas depresivos y otras psicopatologías. Debido a esto, este estudio se centró en analizar los factores predictores de síntomas depresivos y de ansiedad en PVVS. Este es un estudio comunitario (*Índice de Estigma Brasil 2.0*) que incluyó a 1.784 PVVS seleccionadas en siete capitales brasileñas. Los resultados de interés se obtuvieron a partir del *Cuestionario de Salud del Paciente* (CSP) y las covariables fueron aquellas previamente asociadas a resultados desfavorables en materia de salud mental, entre ellas la vulnerabilidad social, la adherencia al tratamiento y el estigma internalizado, entre otras seleccionadas en el estudio *Índice de Estigma*. El estigma internalizado se asoció significativamente con peores resultados de salud mental en todos los modelos (depresión, ansiedad y puntuación total del CSP-4). Otras variables mostraron asociaciones distintas con cada resultado, lo que podría indicar vías de intervención para que los profesionales sanitarios aborden la carga del sufrimiento mental en PVVS. Además, se destacan implicaciones claras para las políticas públicas.

## Introduction

Research from many psychological experiments about the internalization of social norms and the human tendency to conform to external expectations has provided substantial evidence on the impact of stigma on individuals’ attitudes. These insights can be partly applied to the Health field. Discrimination and exclusion related to HIV arise not from the virus itself but from social metaphors and stigma attached to HIV infection and AIDS ^1^. Regardless of others’ empathy, people living with HIV face persistent challenges in overcoming stigma and securing support from family and community. Without engaging in phenomenological debates, these statements simply attempt to bring the attention to the fact that no one can truly comprehend what it is like to be another human being. From a perspective grounded on social determinants of health, this study investigates factors associated with mental health outcomes in people living with HIV/AIDS (PLHA). Although previous research has advanced our comprehension of several factors underpinning psychological distress in PLHA, studies incorporating a myriad of social determinants of health are still needed. This gap might hinder efforts to combat the HIV epidemic given the detrimental effects of social constructs, such as stigmatizing experiences ^2^. 

Substantial advances in biomedical science have also transformed the approach to HIV. In other words, what was once a fatal condition is now, in most cases, considered a manageable, chronic disease. Yet, societal barriers and stigma continue to impact the lives of PLHA ^3^ ^,^ ^4^. Research investigating the links between stigma and mental health has heavily focused on the so-called “key populations”. These groups include gay men, sex workers, individuals whose gender identity differs from that assigned at birth, drug users, and other vulnerable, marginalized groups ^3^. HIV-related stigma among women also has a significant detrimental impact via external and internalized forms of prejudice and bias ^5^ ^,^ ^6^ ^,^ ^7^.

Overemphasizing the significance of stigma within restricted groups might underscore the scientific understanding of broader factors associated with HIV and its related health consequences. Therefore, evidence-based efforts must take the surrounding context into account to properly capture social factors that influence health outcomes. Scholars who adopt this perspective assume that there are no single, universal, and deterministic factors influencing health and behavior.

PLHA suffer various forms of stigma and prejudice throughout their lives. These include hardships within their families, social groups, intimate partners, and interactions with healthcare providers ^8^. Moreover, PLHA commonly face disrespectful treatment and violations of their rights within institutions, groups and other contexts. Due to these overlapping adverse difficulties, many adopt isolation as a strategy to protect themselves from traumatic and hurtful situations ^5^. 

Recent studies examining different types of stigma in PLHA have substantially increased the understanding of direct and indirect effects on depression ^9^ ^,^ ^10^. A systematic review of 83 studies linking stigma to depression revealed that a substantial proportion of them focused on HIV (n = 27), followed by research investigating the role of stigma in depression among sexual and gender minorities ^11^. Internalized stigma can cause psychological distress to the extent of affecting one’s motivation to follow treatment regimens ^10^. Other factors that have been associated with adverse health outcomes include broader forms of discrimination experienced by racial and ethnic groups, refugee or migrant populations, and other specific “key-populations” ^12^ ^,^ ^13^ ^,^ ^14^ ^,^ ^15^. 

Studies highlight the complex interaction of social determinants of health in the context of HIV, emphasizing the need to address not only physical aspects but also psychosocial dimensions that permeate the lives of PLHA ^16^. In fact, the social and emotional withdrawal that occurs in response to the burden of stigma faced by PLHA might be an expression of a coping mechanism. Unfortunately, stigma has been linked to lower treatment adherence ^16^, manifested in behaviors such as missing medications due fear and concerns about having the diagnosis revealed without consent, delays in starting antiretroviral treatment (ART) following diagnosis, and disengagement from healthcare providers ^5^ ^,^ ^6^ ^,^ ^7^. Missing appointments - often resulting from social withdrawal - can negatively impact adherence to ART and substantially increase the risk of acquiring opportunistic infections as individuals disengage from continuous care and social support groups ^8^ ^,^ ^10^ ^,^ ^15^ ^,^ ^16^. Unsurprisingly, evidence links internalized stigma to higher viral loads ^17^ ^,^ ^18^.

Many countries encounter difficulties related to drug resistance in different types of ART regimens due to genetic mutations, as well as HIV-related stigmatization and discrimination that hinder immediate responses ^18^ ^,^ ^19^ ^,^ ^20^. Given these challenges, researchers worldwide have been conducting studies to strengthen preventive measures and uphold commitments to eliminate all forms of stigmatizing experiences for PLHA ^3^. Gathering empirical evidence is crucial to address stigma, particularly to understand its different forms, degrees, correlations, and effects on the lives of those impacted. 

### The present study

Healthcare-related stigma can hinder regular attendance at medical appointments, posing a challenge to the Joint United Nations Programme on HIV/AIDS’ (UNAIDS) objectives for the next five years, specifically: eliminating all HIV-related stigma in healthcare settings and treating 95% of diagnosed PLHA ^21^. Consequently, a cross-national network is conducting studies using the *Stigma Index Brazil 2.0*.

The reviewed literature highlights several factors that can compromise one’s psychological state, including PLHA ^15^ ^,^ ^22^ ^,^ ^23^ ^,^ ^24^ ^,^ ^25^ ^,^ ^26^. Some risk factors may affect individuals differently and indirectly, depending on resources and support available to them. Low education and income, older age, precarious social support, HIV-related stigma, anticipated stigma, and internalized stigma are some examples ^15^ ^,^ ^22^ ^,^ ^23^ ^,^ ^24^ ^,^ ^25^ ^,^ ^26^. 

This study aims to explore the predictors of psychological suffering in PLHA, with particular emphasis on the role of internalized stigma. The relevance of this study is threefold. First, the investigation will test the hypothesis that internalized stigma is significantly linked to depression. Second, the study will not only build upon and complement the existing research but will consider additional outcomes - namely, anxiety and the combined score of the *Patient Health Questionnaire* (PHQ-4) ^27^. Finally, another contribution for stigma researchers lies in the examination of psychometric properties of the PHQ-4 among PLHA in Brazil. To the best of our knowledge, this is the first nationwide study to account for the role of stigma in mental health outcomes among PLHA while also providing support for a proper measurement methodology.

## Method

### Study population and procedures

This community-based study included 1,784 PLHA, recruited from seven Brazilian capital cities. The sample was determined a priori. Participants’ ages ranged from 18 to 76 years (µ = 40.14 ± 12.95). Moreover, individuals reported living with HIV for a mean of 10.61 years (standard deviation - SD = 8.76). Most participants identified as male (n = 1,125; 63.84%), followed by female (n = 566; 32.12%) and transgender/non-binary (n = 71; 4.02%). Furthermore, there was a greater representation of black, mixed-race, yellow, and Indigenous people (n = 1,239; 69.68%). Further demographic and clinical details on this population can be found in the *Executive Summary: Stigma Index for People Living with HIV/AIDS Brazil* ^28^. Under the coordination of researchers from the Rio Grande do Sul Catholic University (PUCRS, acronym in Portuguese), 30 PLHA received training to conduct data collection ^28^.

Non-probabilistic snowball sampling was used to recruit participants, stemming from networks of interviewers located in the seven designated focus cities. Interviewers were individuals living with HIV and were encouraged to identify prospective participants within their peer networks, as well as via various support groups, testing centers, healthcare services, and organizations involved in HIV/AIDS interventions. The selection process aimed to yield a heterogeneous sample, mirroring the strategic priorities of the Brazilian HIV/AIDS response, including representation of black individuals, youth, older adults, and other key populations. Throughout training and data collection, interviewers were instructed to recruit participants from venues commonly frequented by the target demographics, using their own social networks while considering segmentation by race/ethnicity, age, sexual orientation, gender identity, socioeconomic status, substance use, incarceration history, and involvement in sex work. The study was conducted as a community-based survey in which PLHA conducted face-to-face interviews with their peers at locations mutually agreed upon by both interviewer and participant within the targeted urban areas. Inclusion criteria encompassed being an individual living with HIV/AIDS aged 18 years or older, residing in one of the focus cities, and providing informed consent to partake in the research.

### Study variables

The study outcomes include anxiety and depression scores, as well as the composite score of the PHQ-4 ^27^, a brief screening tool designed to assess depression and anxiety, consisting of four items. Responses range from 0 (“not at all”) to 3 (“nearly every day”). Severity can be assessed for each subscale, with scores of 3 or higher suggesting clinically significant symptoms. Overall PHQ-4 scores are defined as normal (0-2 points), mild (3-5 points), moderate (6-8 points), and severe (9-12 points).

Independent variables were extracted from the *Stigma Index 2.0* and demonstrated theoretical and ecological relevance to the study, including demographic information, HIV-related details, indicators of vulnerability, among others (Table 1). These variables are part of a comprehensive list of questions fully described elsewhere ^3^ ^,^ ^27^.

**Table 1:** Association between study variables.

Parameter	1	2	3	4	5	6	7	8	9	10	11	12	13
1. Age													
rho	-												
p-value	-												
2. Years living with HIV													
rho	0.560	-											
p-value	< 0.001	-											
3. Education													
rho	-0.196	-0.125	-										
p-value	< 0.001	< 0.001	-										
4. ART non-adherence													
rho	- 0.118	- 0.016	-0.014	-									
p-value	< 0.001	0.508	0.563	-									
5. ART													
rho	-0.050	-0.037	-0.034	0.021	-								
p-value	0.037	0.121	0.155	0.384	-								
6. Hardship in the previous year													
rho	0.047	0.069	-0.269	0.102	0.023	-							
p-value	0.048	0.004	< 0.001	< 0.001	0.334	-							
7. Ethnic minority													
rho	-0.091	-0.060	-0.001	-0.016	0.018	0.037	-						
p-value	< 0.001	0.012	0.992	0.497	0.452	0.122	-						
8. Religion minority													
rho	0.061	0.040	-0.091	0.018	-0.017	0.104	0.204	-					
p-value	0.011	0.094	< 0.001	0.463	0.486	< 0.001	< 0.001	-					
9. HIV “key population”													
rho	-0.249	-0.214	0.308	-0.013	-0.029	-0.126	0.057	-0.038	-				
p-value	< 0.001	< 0.001	< 0.001	0.583	0.227	< 0.001	0.017	0.109	-				
10. Internalized stigma													
rho	-0.119	-0.160	-0.156	0.227	0.044	0.148	-0.003	0.022	-0.053	-			
p-value	< 0.001	< 0.001	< 0.001	< 0.001	0.066	< 0.001	0.913	0.361	0.027	-			
11. Depression													
rho	-0.066	< 0.001	-0.120	0.127	0.079	0.282	0.028	0.031	-0.009	0.342	-		
p-value	0.005	0.986	< 0.001	< 0.001	< 0.001	< 0.001	0.250	0.202	0.696	< 0.001	-		
12. Anxiety													
rho	-0.016	0.019	-0.137	0.152	0.077	0.247	0.016	0.056	-0.036	0.320	0.606	-	
p-value	0.494	0.426	< 0.001	< 0.001	0.001	< 0.001	0.495	0.021	0.131	< 0.001	< 0.001	-	
13. PHQ-4													
rho	-0.046	0.012	-0.148	0.154	0.085	0.295	0.025	0.046	-0.027	0.366	0.887	0.899	-
p-value	0.055	0.630	< 0.001	< 0.001	< 0.001	< 0.001	0.296	0.056	0.250	< 0.001	< 0.001	< 0.001	-

ART: antiretroviral therapy; PHQ-4: *Patient Health Questionnaire*.

Regarding covariates, the *Internalized AIDS-Related Stigma Scale* (IA-RSS) was used ^29^. This measure consists of six items assessing the internalization of stigma, including feelings of guilt and shame. Responses are given in a dichotomous format, with higher scores indicating greater internalized stigma ^29^. 

### Data analysis

Data were analyzed using the SPSS version 25 (https://www.ibm.com/) and Jasp version 0.19 (https://jasp-stats.org/). Analyses included descriptive statistics such as means (µ), SD, frequencies, and percentages. Inferential methods were used according to the study’s goal, including Spearman’s correlations and regression analyses. Spearman’s correlations were selected because the variables did not follow a normal distribution, as indicated by the Shapiro-Wilk test (p < 0.001). Additionally, confirmatory factor analyses (CFA) were performed to further assess the adequacy of stigma measures used in the study. Alongside the chi-square goodness-of-fit test, the following indices were used to evaluate model fit: Bentler-Bonett normed fit index (NFI), Tucker-Lewis index (TLI), comparative fit index (CFI), root mean square error of approximation (RMSEA), and standardized root mean square residual (SRMR). Interpretations of fit indices strictly followed established guidelines ^30^.

Predictive analyses were conducted for each outcome using linear regression. Independent variables deemed eligible were those associated with any of the outcomes at p < 0.20 in bivariate analyses. The p < 0.20 criterion constitutes a preliminary screening stage intended to optimize the inclusion of potentially relevant variables into the candidate set for multivariate analysis. This approach is widely used in statistical modeling when the goals is to develop a robust predictive model or to adjust for possible biases affecting cross-sectional studies ^31^. 

Standardized and bootstrapped estimates were calculated with respective 95% confidence intervals (95%CI). Assumptions for regression procedures included normality of residuals, assessed via Q-Q scatterplots; homoscedasticity, evaluated by plotting residuals against predicted values ^32^; and multicollinearity, assessed by the inspection of variance inflation factors (VIFs) ^33^. To identify potential outliers, studentized residuals were calculated and plotted. No violations of linear regression assumptions were detected in any of the models.

### Ethical considerations

The research followed all national and international protocols regarding ethics in research involving human beings. Participants signed informed consent forms and were reassured about ethical aspects related to the study. Data collection commenced only after receiving ethical clearance (PUCRS Research Ethics Committee, n. 99716918.5.0000.5336).

## Results

Given the ordinal nature of the items, CFA analyses were conducted using the diagonally weighted least squares (DWLS) estimator. For the PHQ-4, the model demonstrated good fit (χ^2^[1] = 1.64, p = 0.20), with all fit indices falling within optimal ranges (CFI = 0.99; NFI = 0.99; RMSEA = 0.01; SRMR = 0.01; TLI = 0.99). As for the IA-RSS, model fit was acceptable (χ^2^[1] = 16.53, p = 0.02), with additional fit measures (CFI = 0.99; NFI = 0.99; RMSEA = 0.02; SRMR = 0.01; TLI = 0.99).

Subsequently, correlational analyses were conducted (Table 1). Spearman’s coefficients indicated a strong, positive correlation between age and years living with HIV (rho = 0.56, p < 0.001). Conversely, a negative correlation was found regarding age (rho = -0.196, p < 0.001) and internalized stigma (rho = -0.156, p < 0.001). Moreover, internalized stigma was linked with both depression (rho = 0.342, p < 0.001) and anxiety (rho = 0.32, p < 0.001).

Linear regression analyses were performed. Table 2 shows the standardized coefficients and 95%CI for analyses with and without bootstrapping. Internalized stigma was significantly associated with worse mental health outcomes in all models tested. Specifically, a one-unit increase of internalized stigma increased the values of anxiety by 0.30 units, depression by 0.35, and overall mental health by 0.65. Stroger predictors were found in the PHQ-4 model, indicating that patients not currently on ART and those experiencing social vulnerability demonstrated a 1.84 and 1.45 increase, respectively, in the overall PHQ score compared to those on ART and those not facing social vulnerability.

**Table 2:** Predictors of anxiety, depression, and total scores on the *Patient Health Questionnaire* (PHQ-4) among people living with HIV in Brazil.

	B	SE	95%CI	β	p-value	B0	SE	95%CI
Anxiety								
Internalized stigma	0.300	0.030	0.240; 0.350	0.27	< 0.001	0.300	0.030	0.240; 0.350
Hardships in the past year (yes)	0.650	0.090	0.480; 0.820	0.17	< 0.001	0.650	0.090	0.480; 0.820
ART non-adherence	0.280	0.100	0.090; 0.480	0.07	0.005	0.280	0.100	0.090; 0.470
Currently not on ART	0.990	0.380	0.250; 1.740	0.06	0.009	0.990	0.390	0.200; 1.720
Years living with HIV	0.009	0.005	-0.003; 0.020	0.04	0.057	0.009	0.005	-0.002; 0.020
Depression								
Internalized stigma	0.350	0.030	0.300; 0.400	0.32	< 0.001	0.350	0.030	0.290; 0.390
Hardships in the past year (yes)	0.820	0.090	0.650; 0.990	0.22	< 0.001	0.820	0.080	0.660; 0.990
Currently not on ART	0.860	0.370	0.140; 1.590	0.05	0.020	0.860	0.440	-0.050; 1.720
Years living with HIV	0.020	0.006	0.005; 0.030	0.08	0.005	0.020	0.006	0.005; 0.030
Age	-0.009	0.004	-0.020; -0.001	-0.06	0.020	-0.080	0.004	-0.020; 0.001
Key group (yes)	0.130	0.090	0.050; 0.310	0.03	0.153	0.130	0.090	-0.060; 0.310
PHQ-4								
Internalized stigma	0.630	0.050	0.540; 0.720	0.32	< 0.001	0.630	0.050	0.540; 0.720
Hardships in the past year (yes)	1.450	0.150	1.150; 1.750	0.22	< 0.001	1.450	0.150	1.150; 1.750
Currently not on ART	1.840	0.660	0.550; 3.120	0.06	0.005	1.840	0.750	0.270; 3.250
ART non-adherence	0.370	0.170	0.030; 0.720	0.05	0.032	0.370	0.170	0.040; 0.710
Years living with HIV	0.030	0.010	0.006; 0.050	0.07	0.010	0.030	0.010	0.006; 0.050
Age	-0.010	0.007	-0.030; 0.001	0.05	0.076	-0.010	0.007	-0.030; 0.001

95%CI: 95% confidence interval; ART: antiretroviral therapy; B: standardized coefficient; B0: bootstrapped coefficient; SE: standard error.

Note: models fit: F(5,1629) = 52.31, p < 0.001, R^2^ = 0.14 for anxiety; F(6,1626) = 58.30, p < 0.001, R^2^ = 0.18 for depression; F(6,1625) = 64.96, p < 0.001, R^2^ = 0.19 for the PHQ-4.

## Discussion

The goal of this study was to examine factors predicting depressive and anxious symptoms among PLHA. The investigation aimed to contribute to three broader objectives. First, it tested the hypothesis that internalized stigma is strongly associated with depressive symptoms. Second, it aimed to expand and complement previous research by examining additional outcomes, such as anxiety and the composite PHQ-4 score. Finally, the study contributed to the literature by assessing psychometric properties of the PHQ-4 within a sample of PLHA.

As outlined in the literature review, the discussion is also divided into subheadings. However, the authors opted for an interpretative approach to the findings, focusing on underlying mechanisms and practical actions that could be implemented. While comparing specific statistical estimates to those from previous research is a valid and commonly used strategy, we believe that the chosen approach may be more engaging and relevant to the readership of the current journal.

### Results and tentative explanations

Spearman’s correlation coefficients indicated a strong, positive correlation between age and years living with HIV (rho = 0.56, p < 0.001). In contrast, negative correlations were found between age (rho = -0.196, p < 0.001) and internalized stigma (rho = -0.156, p < 0.001), suggesting that higher education levels are associated with lower stigma. Internalized stigma was also positively correlated with depression (rho = 0.342, p < 0.001) and anxiety (rho = 0.32, p < 0.001), suggesting a moderate link with mental well-being. These findings were further supported by regression analyses, which confirmed the bivariate results.

Notably, internalized stigma was found to be a substantial predictor across all models. The standardized coefficients (B = 0.30 for anxiety and B = 0.35 for depression) were statistically significant in both the general and bootstrapped analyses. These results contribute to the extensive body of literature linking HIV and depression, while also expanding the discussion beyond that specific outcome ^17^ ^,^ ^34^ ^,^ ^35^.

As hypothesized, internalized stigma was associated with increased risks for negative mental health outcomes ^22^ ^,^ ^26^ ^,^ ^36^. Another hypothesis - particularly relevant to the national context - established that individuals facing difficulties in meeting basic needs would be more likely to report symptoms of anxiety and depression. Regression analyses also showed that ART-related indicators, such as poor adherence or not being on any type of HIV treatment, increased the chances of poor psychological outcomes, beyond the already well-documented associations with mood disorders. 

By combining the results, several recommendations appear to be relevant. For instance, particular attention should be given to the numerous determinants of adherence at the beginning, resumption, and continuation of treatment ^37^. Healthcare professionals must consistently reinforce the importance of following the prescribed treatment regimen, outlining that the benefits outweigh possible harms ^38^. Combating misinformation and promoting scientific literacy can further boost the success rates of these efforts. 

Likewise, healthcare settings reporting higher ART adherence are usually marked by low levels of prejudice and a high degree of empathy towards stigmas experienced by PLHA ^38^. In this respect, growing evidence suggests that such variables can influence adherence to ART and are closely linked - both implicitly and explicitly - to psychological functioning ^24^ ^,^ ^39^ ^,^ ^40^. From a public health perspective, irregular treatment adherence significantly impacts the effectiveness of combined prevention strategies ^22^ ^,^ ^23^ ^,^ ^24^ ^,^ ^25^ ^,^ ^26^.

Studies have shown that factors contributing to social vulnerability and to fragmented support systems are also statistically linked to psychological suffering in PLHA ^24^ ^,^ ^39^ ^,^ ^40^. In this study, those who responded being unable to fully meet one or more basic needs - such as food or housing - in the year prior to the survey, as well as those with poor or no ART adherence, were at higher risks for psychological suffering. This pattern continues to characterize the lives of many PLHA ^15^ ^,^ ^26^ ^,^ ^41^. Specifically, hardships reported in the past year predicted higher scores for anxiety and depression (B = 0.65 and 0.82, p < 0.001), with even higher estimates for the total PHQ-4 score (B = 1.45, p < 0.001). ART non-adherence, defined as missing doses or not currently being on treatment, was also significantly associated with anxiety and higher PHQ-4 scores (B = 0.28 and 0.37, p < 0.001). 

Moreover, age may also influence key indicators of psychological and physical health among PLHA. Evidence suggests that younger patients often struggle to maintain strict treatment regimens, while living with HIV over extended periods may pose particular challenges for both patients and healthcare providers ^17^ ^,^ ^24^. Data presented in this study suggested that both older age and longer duration of living with HIV were associated with increased impairments in mental health, as measured by the PHQ-4. 

Findings discussed so far underscore the multifaceted nature of mental health challenges faced by this population and will be further discussed next. Healthcare professionals require an understanding of factors that both hinder and facilitate self-care behaviors to effectively the delivery of high-quality care. Factors such as stigma, lengthy and complex treatment protocols, severe side effects, lack of emotional support, experiences of trauma or socioeconomical disadvantage, limited access to healthcare services, low education, mental health issues, cognitive impairments, denial of serological status, racial and ethnic disparities, poor communication with healthcare providers, and misconceptions concerning HIV and its treatment can all lead to adverse outcomes ^5^ ^,^ ^13^ ^,^ ^15^.

### Implications, limitations, and further directions

Both direct and indirect implications for health services and policymakers emerge from this research, corroborating the importance of providing immediate psychological and social support to prevent critical levels of suffering due to stigmatizing experiences ^22^ ^,^ ^24^ ^,^ ^25^ ^26^ ^,^ ^34^. Such interventions could assist in achieving the proposed goals for HIV elimination ^42^. When addressing the forms of stigma experienced by people living with HIV, multifaceted efforts that consider psychological, social, behavioral, and public health determinants align with current competencies and best practices in HIV care ^22^. Additionally, strategies to effectively boost social support and psychological well-being are necessary; however, progress in this area remains limited. Similarly, social protection systems, including livelihood interventions that promote economic empowerment, can also mitigate both the independent and dependent variables identified in this study ^26^. 

Interventions aimed at eliminating stigma at the individual, community, and structural levels are therefore essential. Findings reported here are pivotal for the development of effective programs and policies for PLHA.

It is important to acknowledge limitations associated with observational designs, including potential biases during data collection (i.e., desirability bias). The use of a convenience sample constrains the generalizability of the findings to the broader population living with HIV/AIDS in Brazil. Additionally, the focus on capital cities may neglect the experiences of individuals residing in rural areas or smaller municipalities. Although the PHQ-4 is widely used screening purposes, it may not fully capture all symptoms of anxiety and depression. Nonetheless, a key strength of this study lies in its description of robust psychometric indices for the PHQ-4, supported by a large and nationally representative sample. Once again, implications for policy and practice emerge, such as the routine use of ultra-brief instruments like the PHQ-4 during follow-up consultations. Early preventive and interventional actions could potentially reduce costs associated with referring patients across multiple services. The findings also highlight the need for further investigation into other types of stigma, including anticipated stigma. Longitudinal studies are recommended to investigate causal relationships between variables associated with psychological distress in this sample.

## Conclusion

The internalization of societal stigmas by PLHA is a highly complex process, with significant implications for their self-perception, their experience living with HIV, and associated implications. This, in turn, may perpetuate a negative cycle involving low self-worth, feelings of shame, and avoidant behaviors. Internalized stigma among PLHA poses a substantial barrier to both HIV prevention and treatment efforts. Key results demonstrated that predictors of depression and anxiety involved ART-related indicators, duration of time living with HIV, and markers of social vulnerability.

## Data Availability

The research data are available upon request to the corresponding author.
